# Evaluating the performance of digital surveillance for epidemic-prone diseases in Kwango Province, Democratic Republic of Congo

**DOI:** 10.3389/fpubh.2025.1669745

**Published:** 2025-11-19

**Authors:** Ayuko Hirai, Bernard Silenou, Tommy Bukasa, Baweye M. Barka, François Mwakisenda, Juliane Dörrbecker, Tshotsho Dixon, Aruna Aaron, Mathias Altmann

**Affiliations:** 1 National Institute for Health and Medical Research (Inserm) UMR 1219, Research Institute for Sustainable Development (IRD) EMR 271, Bordeaux Population Health Research Centre, University of Bordeaux, Bordeaux, France; 2 Helmholtz Centre for Infection Research, Braunschweig, Germany; 3 The Alliance for International Medical Action (ALIMA), Kinshasa, Democratic Republic of Congo; 4 Provincial Health Division, Kwango, Democratic Republic of Congo; 5 Ministry of Health, Kinshasa, Democratic Republic of Congo

**Keywords:** digital health technology, eHealth, SORMAS, infectious disease surveillance, epidemic preparedness, Democratic Republic of Congo

## Abstract

**Background:**

Strengthening infectious disease surveillance systems is critical to prevent the spread of diseases, particularly in resource-limited settings. Digital health tools such as the Surveillance Outbreak Response Management and Analysis System (SORMAS) offer real-time reporting and data management. This study assessed the performance of SORMAS in Kwango Province, Democratic Republic of Congo (DRC), focusing on two implementation models: facility-level model, where health workers at health facilities entered data directly into SORMAS, and health zone-level model, where data entry was centralized at the health zone office.

**Methods:**

From July 2022 to December 2024, SORMAS was piloted for case-based reporting of epidemic-prone diseases in the Kenge Health Zone via the facility-level model and in 13 other health zones via the health zone-level model. We evaluated the completeness and timeliness of case-based reporting, as well as concordance with the conventional paper-based weekly epidemiological reports (WERs). SORMAS user characteristics were obtained through a telephone survey.

**Results:**

A total of 2,950 cases were registered in SORMAS between July 2022 and August 2024. The completeness of non-mandatory epidemiological data exceeded 80% across both implementation models. Timely reporting (within 1 day) was significantly greater under the facility-level model (46.0%) than under the health zone-level model (5.0%). SORMAS reported more cases than WERs under facility-level implementation, whereas WERs captured more cases than SORMAS under the health zone-level model.

**Conclusion:**

SORMAS is a viable tool for enhancing disease surveillance in the DRC, particularly when implemented at the health facility level. This pilot demonstrates the potential of digital tools to improve outbreak preparedness and response in resource-limited settings.

## Background

Effective infectious disease surveillance is essential for the early detection of outbreaks and timely responses to prevent the spread of diseases (1). The Integrated Disease Surveillance and Response (IDSR) strategy, developed by the World Health Organization Regional Office for Africa (WHO AFRO), supports African countries in meeting the requirements of the International Health Regulations (IHRs) (2005), which mandate improvements in their capacity to prepare for, detect, and respond to health emergencies (2, 3).

Despite the recognized importance of electronic health (eHealth) tools in modernizing health systems (4–6), the adoption of digital data collection and management tools remains limited in many low-income settings (5, 7–9). This limitation poses significant challenges to an efficient outbreak response. In resource-limited settings, such as the Democratic Republic of Congo (DRC), strengthening disease surveillance systems through digitalization is particularly critical because of the recurrent public health challenges posed by epidemic-prone diseases.

The DRC faces ongoing public health threats due to recurrent epidemics, including Ebola, cholera, measles, yellow fever, and the recent national outbreak of mpox, declared in December 2022 (10–12). However, disease surveillance has traditionally relied heavily on paper-based tools, particularly at the sub-provincial level, which limits the effectiveness of early warning and response systems. Although digital tools such as the Early Warning, Alert and Response System (EWARS) and the District Health Information Software version 2 (DHIS2) Tracker have been deployed in several provinces in recent years, many regions—including Kwango Province—still lack access to digital surveillance tools, and the effectiveness of these systems has not been fully evaluated in the DRC.

The Surveillance Outbreak Response Management and Analysis System (SORMAS) is an open-source tool designed for seamless use on desktop and mobile applications (13). Initially developed in response to the 2014–2015 Ebola outbreak in West Africa (14, 15), SORMAS has since been used globally in approximately 15 countries during the COVID-19 pandemic (16, 17), including Nigeria (18) and Ghana (19), where it has been implemented nationwide. SORMAS enables documenting infectious disease cases, case and contact management, and real-time statistics and data visualizations. The tool offers over 40 disease modules, and its offline functionality allows users to enter data without an Internet connection when using the mobile application, with synchronization occurring once connectivity is restored (13). The objective of this study is to assess the performance of the digital tool SORMAS, piloted for the first time in DRC, by comparing its implementation at the health facility level (facility level) with that at the health zone level in Kwango Province. This assessment aims to provide insights into the potential of digital tools to improve outbreak preparedness and response in resource-limited settings.

## Methods

### Study setting

**Figure 1:**
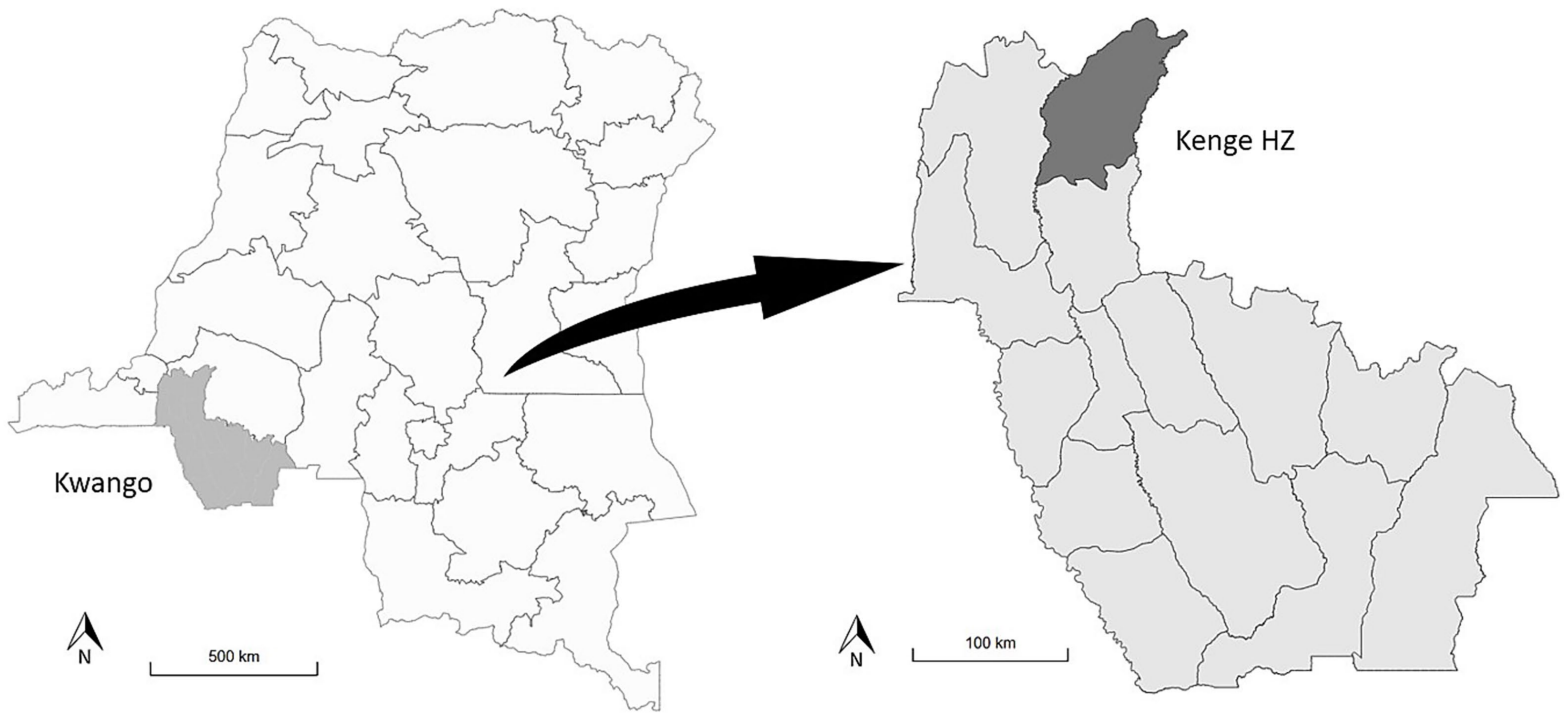
Map of the study area: Kwango Province and Kenge Health Zone, Democratic Republic of Congo. HZ, health zone.

This study was conducted in Kwango Province, which is located in the southwestern region of the DRC. Kwango Province covers an area of 89,974 km^2^ and has an estimated population of 2,863,627 in 2024 (20). The province is divided into 14 health zones (HZs), which are further subdivided into 308 health areas (HAs). Each HA is typically served by a single health center. Kenge, the capital of Kwango Province, is located within Kenge HZ and is further divided into 29 HAs (Figure 1). Kwango Province was selected for the pilot implementation of SORMAS in collaboration with the Epidemiological Surveillance Department of the Ministry of Health. The reasons for selecting this province include its lack of digitalization for case-based surveillance and its geographical proximity to the capital, which facilitates supervision.

In the DRC, to increase the IDSR, weekly epidemiological records (WERs) are collected at two levels within the province: the HA level and the HZ level. WERs consist of aggregated data on 24 diseases and syndromic diagnoses, including the number of reported cases and fatalities on a weekly basis. However, unlike SORMAS, the WER system is not adapted to collect individual data. In each HA, the nursing officers (*infirmiers titulaires*) at health centers are responsible for collecting epidemiological data. There are also nursing officers in every general hospital in each HZ. The nursing officers at both the health centers and general hospitals compile the data weekly and send it to nurse supervisors (*infirmiers superviseurs*) at the central office of the respective HZ. The nurse supervisors aggregate the data from all the nursing officers within the HZ and then send it to the Provincial Health Department.

**Figure 2:**
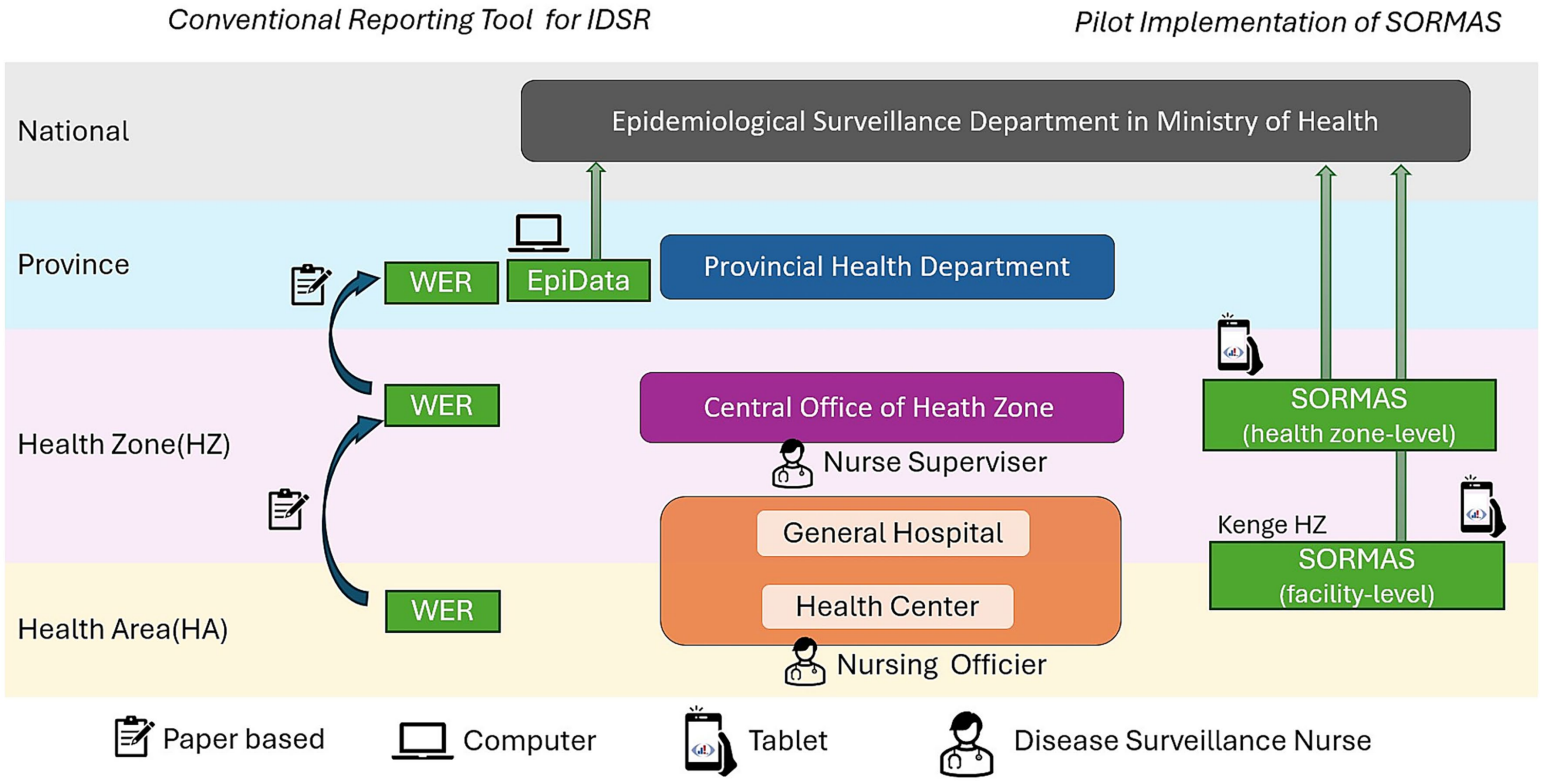
Tools used in conventional surveillance and SORMAS pilot in Kwango Province, July 2022–December 2024. IDSR, Integrated Disease Surveillance and Response; DRC, Democratic Republic of Congo; SORMAS, Surveillance Outbreak Response Management and Analysis System; HZ, health zone; WERs, Weekly Epidemiological Records.

In Kwango Province, this data collection and transmission process relies on paper-based methods. However, in practice, these paper-based WER reports are often transmitted by sending photographs of the WER forms via WhatsApp (21) rather than the physical form. Once the data are collected from the nurse supervisors, WERs are entered into a digital database (EpiData) (22) by the surveillance officer at the Provincial Health Department and subsequently shared with the National Epidemiological Surveillance Department at the Ministry of Health (Figure 2).

### Study design

This study was conducted from July 2022 to December 2024 as part of the pilot implementation of SORMAS in Kwango Province. The implementation of SORMAS was carried out at two levels (Figure 2):

- *Facility level (Kenge HZ)*: SORMAS was piloted in 29 health centers (HAs) and 1 general hospital located in Kenge HZ.
- *Health zone level (other HZs)*: SORMAS was piloted in the central office of 13 other HZs across Kwango Province.

The primary users of SORMAS were 30 nursing officers responsible for surveillance in Kenge HZ and 14 nurse supervisors located at the central offices of each health zone, including Kenge HZ. In Kenge HZ, the nurse supervisor was not the primary person entering case-based data into SORMAS; instead, his role focused on overseeing the work of nursing officers. Tablets preinstalled with SORMAS were provided to primary users, whereas surveillance officers at the provincial and national levels accessed SORMAS via their desktop computers.

Throughout the study period, the conventional aggregated report, WERs, continued to be used in parallel. To compare the two implementation models, statistical analyses were performed using R (version 4.4.1, R Foundation for Statistical Computing, Vienna, Austria). A *p*-value of less than 0.05 was considered statistically significant.

### SORMAS user characteristics

#### Data collection

A telephone survey of SORMAS users was conducted mid-term of the pilot, targeting 44 SORMAS users (30 nursing officers and 14 nurse supervisors). The data collected by the survey included sociodemographic information (such as sex, age, and professional experience) and Internet access. The survey was carried out in April 2023 by a Congolese interviewer using French and local languages.

#### Data analysis

The users were categorized into two groups, facility level and health zone level, based on the implementation model in their health zone. Data from the telephone survey were compiled into an Excel file and used for analysis. Descriptive statistics were applied to summarize the characteristics of SORMAS users, including their Internet access patterns. To compare the two groups, chi-squared tests were performed for categorical variables such as sex, age group, professional experience, and Internet access.

### Performance indicators for digital tool evaluation

#### Data collection

The SORMAS data used in this study were case-based and covered the period from July 2022 to August 2024. These data included 11 diseases and syndromes: measles, influenza, mpox, yellow fever, meningitis, acute flaccid paralysis, COVID-19, rabies, Guinea Worm, “not yet defined,” and “others”.

For the comparison with the conventional reporting system, WER data from Kwango Province for the same period as SORMAS (July 2022 to August 2024) were used. The WER system provides aggregated data on epidemic-prone diseases and syndromes, and its database contains the number of cases and deaths across each age category (neonate, less than 11 months, 12–59 months, 5–15 years, and more than 15 years). These data are organized by health zone but are not disaggregated at the health area level.

#### Data analysis

The case-based data in SORMAS were assessed on the basis of the three attributes: completeness, timeliness, and reported case numbers. Each attribute is defined and analyzed as follows.

##### Completeness

We compared the comprehensiveness of the case-based surveillance data captured in SORMAS between the two implementation models. For this, we first assessed the completeness of the epidemiological information for each individual record. Subsequently, we sorted the records according to the two implementation models, facility level and health zone level, and performed comparative analyses using chi-squared tests.

Completeness was assessed by studying data fields that are not mandatory for creating a case in SORMAS. Certain data fields are not mandatory for case registration, whereas several fields are mandatory. Examples of these fields are as follows:

- *Not mandatory* var*iables*: age, patient’s address, symptom onset date, pregnancy status, and vaccination status
- *Mandatory variables*: disease, date of report, case classification, and outcome

In this study, the following four fields were analyzed: age, patient’s address, pregnancy status, and vaccination status, as this information is essential for an effective outbreak response (23). The vaccination status was analyzed specifically for measles cases, as measles was the most frequently reported disease, and some other diseases—such as influenza—were not part of the national vaccination schedule and were thus not applicable to this analysis.

Each variable was considered complete if a valid and non-missing entry was present in the record; otherwise, it was marked as incomplete. Completeness was calculated as the percentage of cases with complete data divided by the total number of cases reported in SORMAS:

$$\text{Completeness}(\%)=\frac{\left(\text{number of cases with complete data}\right)}{\left(\text{total number of cases in SORMAS}\right)}\times 100\%$$

The denominators used for pregnancy status and vaccination status were the number of female patients aged over 15 years and the total number of measles cases, respectively.

##### Timeliness

This analysis aimed to compare the notification delay within SORMAS between facility-level and health zone-level implementation models. The notification delay was defined as the difference between the date of report and the date of data entry into SORMAS, with the results expressed in whole days. The date of the report was the date of the initial consultation or investigation. This differs from the date of data entry, which was automatically generated by the SORMAS platform when the case was registered in the system. Only cases with plausible data for both dates were included in the analysis. The timeliness was categorized into three intervals—0–1 day, 2–7 days, and more than 7 days (>7 days)—and expressed as cumulative percentages of cases reported within each time threshold. Comparisons between facility-level and health zone-level models were conducted via the chi-squared test.

##### Reported case numbers

We compared the number of case-based data reported through SORMAS to the aggregated case numbers reported by the conventional system, WERs. Among the 11 epidemic-prone diseases captured in SORMAS, three were selected for analysis, namely measles, yellow fever, and mpox, as these are consistently reported in both data sources. The comparison was stratified by the two implementation models. For each disease, the total number of cases recorded in SORMAS and WERs over the same reporting period was extracted and compared.

### Ethics approval and consent to participate

This study received ethical approval from the National Health Ethics Committee of the Democratic Republic of Congo (Approval Number: No 440/cnfs/rn/pmmf/2023). Prior to the telephone survey, all participants were informed about the study and provided oral informed consent. For the analysis of case-based surveillance data, only anonymized and de-identified records were used, with no access to personally identifiable information. All methods were carried out in accordance with the ethical principles of the Declaration of Helsinki.

## Results

### Characteristics of SORMAS users in Kwango Province

Among the 31 SORMAS users of the facility-level implementation model (30 nursing officers and 1 nurse supervisor), 26 (83.9%) were reachable and participated in the survey, whereas 12 out of the 13 users of the health zone-level implementation model (92.3%) were successfully contacted. The remaining users could not be reached by telephone during the 4-day survey period.

The majority of participants in both groups were male (facility level: 84.6%, health zone level: 91.7%) and had extensive professional experience as nurses (facility level ≥10 years: 88.5%, health zone-level ≥10 years: 83.3%). The majority of participants were aged 40 years or older (facility level: 80.0%, health zone level: 76.9%). No significant differences were observed between the two groups in terms of sex, age, or professional experience (*p*-values > 0.05). Half of the respondents reported having good Internet access (available always or most of the time); however, approximately half of the participants (facility level: 42.3%, health zone level: 50.0%) had only rare access to the Internet (Table 1).

**Table 1:** General characteristics and internet access of SORMAS users in Kwango Province, April 2023.

Characteristics	Facility level (Kenge HZ)	Health zone level (other HZs)	*p*-value
	*N* = 26	*N* = 12	
Nursing officer/nurse supervisor	25/1	0/12	
Sex			0.935
Female	4(15.4%)	1 (8.3%)	
Male	22 (84.6%)	11 (91.7%)	
Age			0.847
<30 years	1 (3.8%)	0 (0.0%)	
30–40 years	5 (19.2%)	2 (16.7%)	
40–50 years	10 (38.5%)	6 (50.0%)	
>50 years	10 (38.5%)	4 (33.3%)	
Professional experience			0.573
0–5 years	1 (3.8%)	0 (0.0%)	
5–10 years	2 (7.7%)	2 (16.7%)	
>10 years	23 (88.5%)	10 (83.3%)	
Internet access			0.906
Always	5 (19.2%)	2 (16.7%)	
Most of the time	10 (38.5%)	4 (33.3%)	
Rarely	11 (42.3%)	6 (50.0%)	

SORMAS, the Surveillance Outbreak Response Management and Analysis System; HZ, health zone.

### Case-based data reported

From July 2022 to August 2024, a total of 2,950 case-based data entries were recorded in the SORMAS system: 1,103 (37.4%) cases from Kenge HZ, where SORMAS was piloted at the facility level, and 1,847 cases from the other 13 HZs in Kwango, excluding Kenge. Among these, 1,117 cases (37.8%) were measles, 270 cases (9.2%) were mpox, and 221 cases (7.5%) were yellow fever.

### Data completeness for the two implementation models

The completeness of epidemiological data, which was not mandatory for case registration in SORMAS, was globally high in both implementation models (more than 80%). For variables such as age, patient address, and pregnancy status, completeness was significantly greater under the facility-level implementation model, where nursing officers at individual health facilities were responsible for data entry. Vaccination status also showed higher completeness under the facility-level model, although the difference was not statistically significant when compared with the health zone-level approach. In contrast, symptom-related data were more complete under the health zone-level implementation model (Table 2).

**Table 2:** Completeness and timeliness of case-based data entry in SORMAS, in the Kwango Province, July 2022–August 2024.

Attributes	Overall	Facility level (Kenge HZ)	Health zone level (other HZ)	*p*-value
Completeness of information				
Age	2,728/2,950 (92.3%)	1,001/1,103 (90.8%)	1,627/1,847 (88.1%)	0.0289
Patient address	2,713/2,950 (92.0%)	1,081/1,103 (98.0%)	1,632/1,847 (88.4%)	<0.001
Pregnancy	373/419 (89.0%)	167/179 (93.3%)	206/240 (85.6%)	0.023
Vaccination status (measles)	1,011/1,117 (90.5%)	400/437 (91.5%)	611/680 (89.9%)	0.406
At least one symptom	2,665/2,950 (90.3%)	925/1,103 (83.9%)	1,740/1,847 (94.2%)	<0.001
Symptom onset date	2,607/2,665 (97.8%)	901/925 (97.4%)	1,706/1,740 (98.0%)	0.347
Timeliness of data entry				
0–1 days	599/2,925 (20.4%)	507/1,090 (46.5%)	92/1,835 (5.0%)	<0.001
2–7 days	960/2,925 (32.8%)	290/1,090 (73.1%)	670/1,835 (41.5%)	<0.001
>7 days	1,366/2,925	293/1,090	1,073/1,835 (98.8%)	

SORMAS, the Surveillance Outbreak Response Management and Analysis System; HZ, health zone.

### Timeliness of data entry for the two implementation models

Among the 2,950 registered cases in SORMAS, 25 cases (13 at the facility level and 12 at the health zone level) were excluded from the analysis because at least one of the required dates was missing or invalid. For the facility-level model, 46.0% of the cases were registered in SORMAS within 1 day of consultation or investigation, and 72.3% of the cases were registered within 7 days. In contrast, the health zone-level model resulted in considerably slower reporting, with only 5.0% of cases registered within 1 day and 41.3% within 7 days (Table 2). One health zone in particular experienced a substantial delay, registering 0.6% of cases within 1 day and 8.6% within 7 days. However, three other health zones demonstrated relatively rapid reporting, with over 70% of cases registered within 7 days—comparable to the performance observed for the facility-level model.

### Reported case numbers in SORMAS and in the conventional system WERs

**Figure 3:**
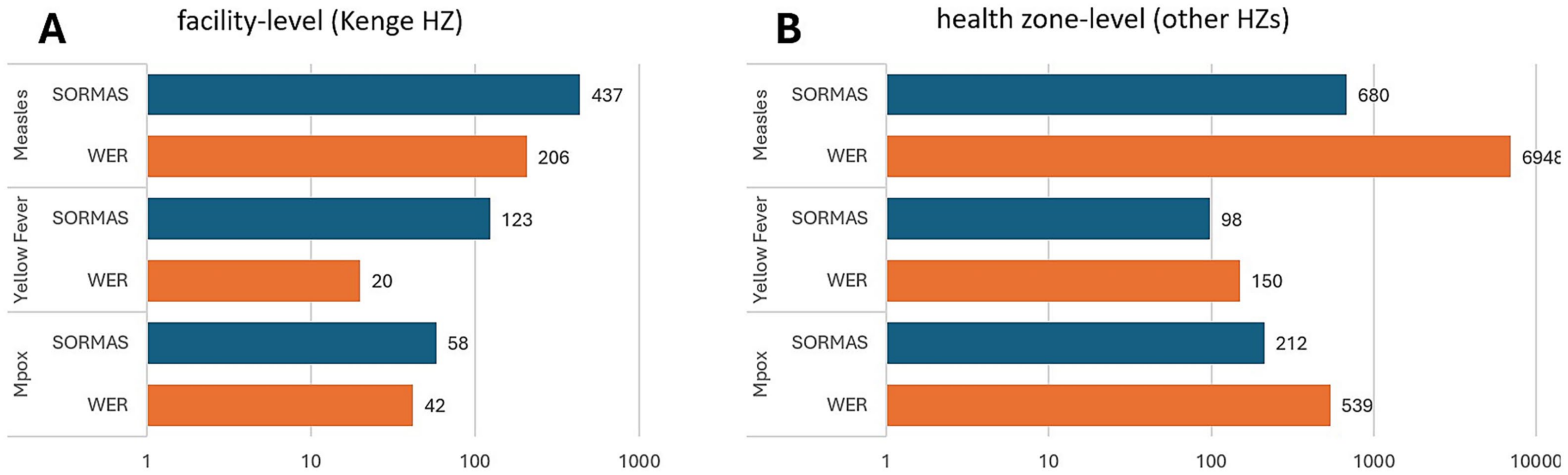
Number of three epidemic-prone disease cases reported in two surveillance systems, in Kwango Province. **(A)** Facility-level model [in Kenge Health Zone (HZ)], where health workers at health facilities entered data directly into SORMAS. **(B)** Health zone-level model (other 13 HZs), where data entry into SORMAS was centralized at the health zone office. SORMAS, Surveillance Outbreak Response Management and Analysis System; WERs, Weekly Epidemiological Records.

In Kenge HZ, where SORMAS was implemented at the health facility level, the system reported a greater number of cases for all three diseases (measles, yellow fever, and mpox) than WERs. In contrast, in other health zones where SORMAS was implemented only at the health zone level, WERs reported a greater number of cases (Figure 3).

Specifically, for measles, SORMAS reported approximately twice as many cases (437 cases in SORMAS vs. 206 cases in WERs) with health facility-level data collection. In contrast, WERs reported approximately 10 times more cases (680 cases in SORMAS vs. 6,948 cases in WERs) with health zone-level data collection. Similarly, SORMAS recorded approximately six times more yellow fever cases than WERs under the health facility-level model (123 cases in SORMAS vs. 20 cases in WERs), whereas WERs reported 1.5 times more cases than SORMAS under the other health zone-level model (98 cases in SORMAS vs. 150 cases in WERs).

## Discussion

This study assessed the pilot implementation of the digital surveillance tool SORMAS and its impact on improving data collection in Kwango Province, DRC. Two implementation models were compared: a facility-level model, where nursing officers at health facilities directly entered data into SORMAS (piloted in Kenge HZ), and a health zone-level model, where nurse supervisors compiled and entered data at the zone level (applied in 13 other HZs).

Overall, the completeness of epidemiological data in SORMAS was high across both models (>80%), indicating that the data collection was comprehensive and allowing for a detailed characterization of an epidemic. For demographic fields, completeness was higher under the facility-level model. However, symptom-related data were more complete under the health zone-level model. This may be explained by a filtering process during data transmission from health facilities to the central health zone office, where cases without clearly defined symptoms might be excluded. This high completeness of data also suggests that SORMAS is user-friendly and facilitates consistent data entry (17).

In terms of timeliness, the facility-level model significantly outperforms the health zone-level model. Approximately 46.0% of the cases were entered into SORMAS within 1 day in Kenge HZ; only 5.0% met this timeframe under the health zone-level implementation model. Timely data entry enables real-time alerts to higher administrative levels and supports the “7-1-7” goal for timely outbreak notification (24, 25). While several HZs achieved comparable reporting rates within 7 days, their reporting performance within 1 day remained far below that of the facility-level model. For example, even the best-performing nurse supervisors accounted for only 18.7% of the cases reported within 1 day. In contrast, WER data collection occurs only once a week and requires aggregation at the health zone and provincial levels, introducing additional delays before the information becomes available at the national level, whereas SORMAS data are transmitted directly to the national level in a timely manner. These differences are largely explained by variations in workflow. In Kenge HZ, nursing officers had direct access to consultation registers and could enter data immediately. In contrast, the health zone-level model requires nurse supervisors to first collect information from multiple facilities. This process leads to delays, communication gaps, and incomplete data transfers.

The total number of reported cases also varied depending on the implementation models: Under the facility-level model, SORMAS recorded more cases than the conventional reporting system WERs, suggesting improved case capture. However, under the health zone-level model, WERs reported more cases than SORMAS, and this discrepancy was significant during the large-scale measles epidemic. This indicates that case-based data entry was challenging for users, as entering numerous individual records is time-consuming, especially during outbreak periods. In Kwango Province, under intensive interventions, a simplified line-list approach and tally sheets were used for data collection, and the consultation record was not always used. While SORMAS includes a function to enter data in a simple line list, this feature was not used in practice during the pilot. It is important to note that, although the results suggest that WERs could better capture the magnitude of the outbreak, they provide limited individual case information, restricting their usefulness for epidemiological analysis.

Furthermore, SORMAS data entry in Kenge HZ likely benefitted from greater flexibility: While WER data must be submitted by Monday from each health facility and are excluded if delayed, SORMAS allows retrospective data entry. This flexibility could explain why the total case count recorded in SORMAS surpassed that in WERs, despite the additional effort required for individual data entry.

These findings indicate that decentralizing digital surveillance at the health facility level can improve both the speed and comprehensiveness of data reporting. Nevertheless, challenges remain. Some health facilities reported very few cases in the study. The workload associated with digital surveillance—including the need to maintain mobile devices and reliable Internet access—was substantial. We recognize that short-term pilot projects may not be sustainable and, in some cases, could risk disrupting existing reporting systems. To ensure continuity of routine surveillance, conventional systems (WERs) were maintained in parallel while piloting SORMAS. The results of the pilot in Kwango Province have generated interest from the Public Health Emergency Operations Center of the DRC in using SORMAS for epidemic response, including for mpox. The Ministry of Health also remains supportive of expanding SORMAS to other areas; however, due to financial constraints, further rollout has been temporarily put on hold following the completion of this pilot. To support the sustainable use of digital tools such as SORMAS, future strategies could explore cost-effective approaches, such as sentinel site surveillance or the shared use of mobile devices among various health initiatives that use digital tools and operate within the same region (26, 27).

It is also worth mentioning the underutilization of SORMAS for laboratory data. During the study period, very few laboratory results were entered into the system. In Kwango Province, laboratory testing capacity is limited, and samples from the province often have to be sent to the central laboratory in Kinshasa. Geographic and logistical barriers caused delays or prevented the transmission of results. While SORMAS enables direct entry of results by laboratory technicians, this requires active collaboration from the laboratories. Although our pilot did not address these aspects, strengthening the laboratory component could enhance outbreak confirmation and response times (28). Similarly, improving the effectiveness of digital tools—through better integration into existing health systems and capacity building for healthcare workers (5, 17, 29)—remains an important area for future consideration.

Several study limitations should be acknowledged. First, the telephone survey used to assess Internet access among SORMAS users may have introduced bias, as those unreachable by phone may also have had limited or no Internet access. Second, implausible data were excluded from the timeliness analysis. Although the amount of excluded data was small, this exclusion may have introduced a selection bias. Third, no financial incentives were provided to SORMAS users, and the traditional surveillance system remained in place throughout the project. This meant that the introduction of SORMAS added to existing workloads without compensation, likely resulting in demotivation and lower user engagement—factors that could have negatively affected both performance and data quality (30). Another key limitation was the absence of a functional case-based reporting system for comparative evaluation. Since individual case reports (line lists) are typically available only during outbreaks, we relied on weekly aggregate data from WERs as a proxy for comparison. While this approach allows for some level of evaluation, it may not fully capture the accuracy needed for robust performance assessments.

Finally, due to financial constraints, the facility-level model was piloted in only one health zone—Kenge, the provincial capital—which benefits from better infrastructure, Internet access, and supervision. In Kenge HZ, direct supervision of SORMAS users was possible through occasional field visits and during group meetings. However, owing to the large size of Kwango Province, in-person supervision was not feasible in other HZs. Some nurse supervisors were located more than 1,000 km from the provincial capital, making remote supervision—via phone or WhatsApp—the only viable option. These factors may have introduced bias in favor of the facility-level model. To ensure more generalizable results, future evaluations should include multiple health zones with varying geographical, infrastructural, and operational contexts.

## Conclusion

In conclusion, this study demonstrates the potential of the digital tool SORMAS to enhance disease surveillance and enable faster, more effective responses to both ongoing and emerging health threats by improving the timeliness, completeness, and reported case numbers. The results also indicate that, owing to its user-friendly design and open-source architecture, SORMAS has potential applicability in other resource-limited contexts facing similar challenges in outbreak management. SORMAS can play a critical role in meeting global health targets such as the 7-1-7 goal, and it has the potential to serve as a unified tool for all surveillance activities, eliminating the need for duplicative reporting systems such as line lists (during epidemics) and separating weekly reports for IDSR and DHIS2.

However, several challenges remain, including addressing logistical barriers, optimizing cost-effectiveness, and fostering collaboration with laboratories. These issues require continuous attention and strategic investments to ensure the sustainable and scalable implementation of digital surveillance tools. Future research should focus on evaluating the long-term impact of SORMAS and similar digital tools on disease surveillance and outbreak response in resource-limited settings, with particular emphasis on the workload, cost and financial implications of large-scale implementation, and sustainability.

## Data Availability

The raw data supporting the conclusions of this article will be made available by the authors, without undue reservation.
